# Distinguishing between Contact and Administration of Heroin from a Single Fingerprint using High Resolution Mass Spectrometry

**DOI:** 10.1093/jat/bkz088

**Published:** 2019-11-04

**Authors:** Catia Costa, Mahado Ismail, Derek Stevenson, Brian Gibson, Roger Webb, Melanie Bailey

**Affiliations:** 1 Ion Beam Centre, University of Surrey, Surrey GU2 7XH, UK; 2 Department of Chemistry, University of Surrey, Surrey GU2 7XH, UK; 3 Forensic Science Ireland, Garda Headquarters, 8 Phoenix Park, Dublin DO8 HN3X, Ireland

## Abstract

Fingerprints have been proposed as a promising new matrix for drug testing. In previous work it has been shown that a fingerprint can be used to distinguish between drug users and nonusers. Herein, we look at the possibility of using a fingerprint to distinguish between dermal contact and administration of heroin. Fingerprint samples were collected from (i) 10 patients attending a drug rehabilitation clinic, (ii) 50 nondrug users and (iii) participants who touched 2 mg street heroin, before and after various hand cleaning procedures. Oral fluid was also taken from the patients. All samples were analyzed using a liquid chromatography—high resolution mass spectrometry method validated in previous work for heroin and 6-AM. The HRMS data were analyzed retrospectively for morphine, codeine, 6-acetylcodeine and noscapine. Heroin and 6-AM were detected in all fingerprint samples produced from contact with heroin, even after hand washing. In contrast, morphine, acetylcodeine and noscapine were successfully removed after hand washing. In patient samples, the detection of morphine, noscapine and acetylcodeine (alongside heroin and 6-AM) gave a closer agreement to patient testimony on whether they had recently used heroin than the detection of heroin and 6-AM alone. This research highlights the importance of washing hands prior to donating a fingerprint sample to distinguish recent contact with heroin from heroin use.

## Introduction

The detection of illicit drugs in fingerprint samples has been widely reported, and there is a growing body of evidence to support the concept that the detection of drug compounds and their metabolites in fingerprint samples can be used to show that a donor has either consumed or handled illicit drugs (1–17). There are two different application areas in which this information might be used—in forensics, where the uncontrolled deposition of a finger at a crime scene leaves a so-called “fingermark” (18, 19), or in drug testing, where a fingerprint sample can be donated under controlled conditions by a known donor (3).

For forensic applications, knowledge that a fingermark has been deposited by someone who has either touched or ingested illicit drugs may help law enforcement authorities to gain a profile of an unknown suspect, perhaps if the fingermark is smudged or the offender is not listed on the fingerprint database (20, 21). In this case, it may be sufficient just to know that a suspect has been in the presence of illicit drugs above environmental levels—analogous to the way that gunshot residue evidence is used to demonstrate only that a suspect has been in the vicinity of a shooting incident (22).

Conversely for clinical drug testing applications, situations may arise in which the testing authority must ensure that administration of a drug can be distinguished from dermal contact (23). We have shown in previous work that provided the fingers of donors are washed prior to deposition of a sample, the levels of cocaine and heroin detected in the fingerprints of drug users generally exceed that of “normal” (based on donors from at a UK university) environmental levels, enabling a cut-off level to be set up to distinguish drug use from environmental contact (4). Within this framework, we have also shown that shaking hands with a drug user does not give a false positive result. Therefore, environmental contamination of cocaine and heroin do not appear to create a problem for fingerprint-based drug testing provided that samples are donated and handled appropriately.

However, for certain scenarios (for example determining whether a user was under the influence of a substance while driving) it may be necessary to distinguish administration of a substance from recent contact *above* normal environmental levels. Our previous work comprised only a small set of donors (*n* = 50) from one geographic location and so is unlikely to be universally applicable, as highlighted by a recent letter to Clinical Chemistry (24).

In previous publications (2, 3, 7, 9, 11), it has been assumed that drug metabolites can be used to imply drug administration (as opposed to contact), but so far the only experimental data to support this assumption is detection of drug metabolites (e.g., 6-AM, the heroin metabolite) in fingerprints following administration of drugs. There are a number of publications that have reported detection of substances after contact with a drug (5, 6, 17), but none have searched for drug metabolites in a fingerprint following drug contact.

In this study, we explore differences in fingerprints produced after direct and indirect contact with street heroin, following hand washing and wiping. We compare this with fingerprint samples (10 donors) collected from patients at a drug rehabilitation clinic and a population (50 donors) of nondrug users. All samples were analyzed using the liquid chromatography (LC–MS) method of our previous work that was validated for heroin and 6-monoacetylmorphine (6-AM) (4). Retrospective analysis of the dataset was also carried out to explore the presence of a wider range of analytes, shown by previous studies to be relevant to heroin use (25, 26). These included the alkaloids codeine and noscapine which can be found in opium poppy and therefore street heroin, as well as the acetylated derivative, acetylcodeine and the metabolite morphine.

## Experimental

### Materials

Drug standards (heroin, 6-acetylmorphine (6-AM), heroin-d_9_ and 6-acetylmorphine-d_3_ (6-AM-d_3_)) were prepared from certified reference materials (Cerilliant). Optima™ LC–MS grade solvents (methanol (MeOH), dichloromethane (DCM), acetonitrile (ACN) and water (H_2_O) were used to prepare all solutions and solvent mixtures (Fischer Scientific). Formic acid was added to the mobile phase at 0.1% (v/v) (Fisher Scientific).

Heroin seized by Irish police and stored at Forensic Science Ireland (FSI) was used for the drug contact experiments. A sample from the drug seizure had been previously analyzed by FSI’s standard analysis protocol using gas chromatography–mass spectrometry (GC–MS) and had been found to have a heroin purity of 11% (Supplementary Data Figure 1). The protocol included the following target substances: phenacetin, caffeine, 6-AM, heroin (diacetylmorphine) and noscapine.

### Sample collection

Fingerprints were collected on 2 × 2 cm squares of Whatman 1-Chr grade chromatography paper, with a single fingerprint collected per sample. Fingerprint samples were collected using kitchen scales (Sainsbury’s Color) to measure the pressure applied during collection (800–1,200 g for 10 seconds).

A favorable ethical opinion for collection and analysis of samples was received from the National Research Ethics Service (NRES—REC reference: 14/LO/0346). Sample collection was carried out in accordance to the relevant guidelines, and informed consent was taken from all participants.

#### Fingerprints produced after drug administration

Fingerprints were collected from individuals (*n* = 10) seeking treatment at drug rehabilitation clinics and testified taking either heroin or cocaine in the last 24 hours. A fingerprint was collected from each finger of the right hand. Participants were instructed to wash their hands thoroughly with soap and water and then wear nitrile gloves for 10 minutes to induce sweating. This was followed by removal of the gloves and finally depositing fingerprint samples. The same process was used to collect fingerprint samples from the right thumb and right index finger from 50 participants who testified not to be drug users.

Corresponding oral fluid samples were collected using a Quantisal™ (Alere™) collection device. Analysis of the oral fluid samples was carried out at Claritest (Norwich, UK). Claritest screening uses immunoassay testing followed by liquid chromatography–tandem mass spectrometry (LC–MS-MS) quantitation if screening is positive (Private Communications with Claritest, Norwich, UK).

#### Fingerprints produced after drug contact

**Figure 1:**
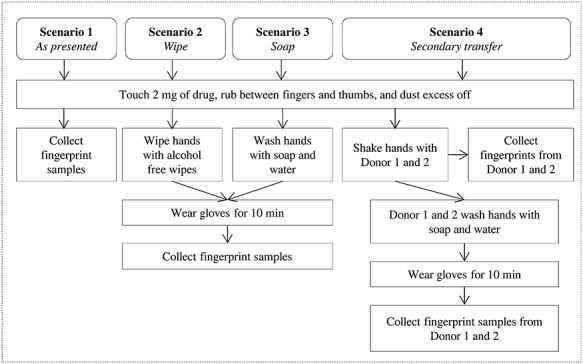
Fingerprint collection procedure used at FSI to determine the presence of drugs (heroin) after contact, washing hands with soap and water, wiping hands with alcohol free wipes and secondary transfer (through shaking hands).

An additional group of participants, who testified to be non-drug users participated in an experiment to generate fingerprints after contact with heroin. Four scenarios were devised to test for the presence of heroin and its metabolites in fingerprints after dermal contact with the parent drug, as illustrated in Figure 1. In Scenario 1, fingerprints were collected from the right thumb, index, middle and ring fingers of three participants after direct contact with 2 mg of heroin (no gloves were worn). In Scenarios 2 and 3, 2 mg heroin was handled by three participants followed by wiping hands with alcohol-free wipes (Scenario 2) or washing hands thoroughly with soap and water (Scenario 3). Following hand cleaning, the participants (*n* = 3) were asked to wear nitrile gloves for 10 minutes before fingerprint samples were collected.

In Scenario 4, a first volunteer touched 2 mg heroin, and hands were shaken with participants 1 and 2. Fingerprint samples were then collected from participants 1 and 2. Another set of fingerprint samples was collected from participants 1 and 2 after washing hands with soap and water and after wearing gloves for 10 minutes, to evaluate the presence of drugs after secondary transfer and hand washing. Samples were given unique identifiers referring to the scenario number (e.g., Scenario 1 = S1) and participant number (e.g., participant 1 = P1) such as S1P1.

### Methods

The fingerprint samples were placed in a 2 mL Eppendorf microcentrifuge tube, with 1.5 mL of 10% DCM in MeOH. The tube was then centrifuged for 2 minutes (at 9.5 centrifugal force). The solvent extract was evaporated to dryness under a stream of nitrogen at room temperature (20°C) and reconstituted in 100 μL of a solution containing 95:5 H_2_O/ACN + 0.1% formic acid + 50 ng/mL heroin-d9 and 6-monoacetylmorphine-d3, before being vortexed and transferred to a 300 μL glass micro-insert vial, with 5 μL injected onto an LC–MS-MS system.

Chromatographic separation was performed on a Thermo Scientific™ Ultimate3000 UHPLC system. Separation was performed on a Kinetex XB-C18 column (100 × 2.1 mm, 5 μm) operated at 30°C at a flow rate of 0.25 mL/min. Gradient analysis was performed with an initial mobile phase of 95:5 H_2_O:ACN (0.1% formic acid), increasing to 80:20 ACN:H_2_O (0.1% formic acid) over 2 minutes, constant for 0.5 minutes before returning to the initial mobile phase composition. The samples were introduced to a Thermo Scientific™ Q-Exactive Plus Orbitrap™ mass spectrometer using the standard electrospray interface with a capillary temperature of 320°C, spray voltage 3 kV and S-lens RF level set at 50%. Positive mass spectra were acquired in full scan mode within a range of *m/z* 50–500 at a mass resolution of 70,000 at *m/z* 200 (AGC target 10^6^ ions and maximum inject time of 200 ms).

#### Method validation

Our previous publication (4) details the method validation that was carried out for heroin and 6-AM, which gave a limit of detection of 40 pg, precision better than 1% and *R*^2^ = 0.9995 in a range 500 pg–10 ng. Although a matrix effect (signal enhancement) for both heroin and 6-AM was observed in the presence of a fingerprint, it varied less than 5% between fingerprint donors.

#### Data processing

Retrospective analysis of the dataset was carried out to explore the presence of codeine, noscapine, acetylcodeine and morphine. Each analyte was assigned based on the *m/z* ratio of the [M + H]^+^ ion observed by inspection of the high resolution mass spectrum (acceptance criterion of +/−5 ppm mass deviation from theoretical *m/z* value) and comparison with samples extracted from blank paper. Extracted ion chromatograms were then produced for relevant analytes. The retention time and accurate monoisotopic masses (see Supplementary Data Table 1) of the compounds were used to create a processing method for rapid data analysis using TraceFinder v4.1 (Thermo Scientific, Bremen). Retrospective analysis of high resolution mass spectrometry (HR-MS) data is described elsewhere (27). Peak assignment was confirmed by comparison with the GC–MS analysis performed at FSI of street heroin. All analyte signals were normalized to the heroin-D9 signal to obtain a ratio analyte:internal standard (A/IS).

## Results

Consistent with our previous study (4), the data from fingerprints of 50 nondrug users (see Supplementary Data Figure 2) show that none of the analytes of interest (heroin, 6-AM, codeine, noscapine, acetylcodeine or morphine) are detected in fingerprints collected after hand washing.

**Figure 2:**
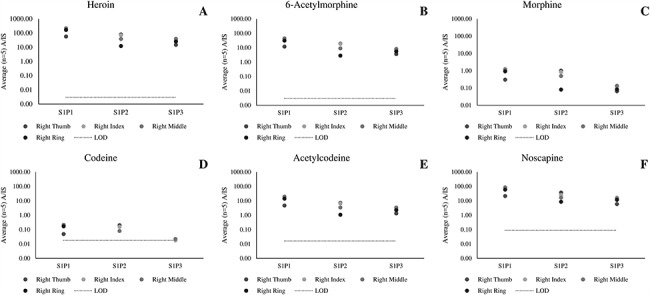
Average (*n* = 5 repeat injections) A/IS ratio of (A) heroin, (B) 6-acetylmorphone, (C) morphine, (D) codeine, (E) acetylcodeine and (F) noscapine measured using LC–HR-MS for fingerprint samples collected after dermal contact with 2 mg of street heroin (Scenario 1).

Figure 2(A–E) presents the ratio A/IS for the analytes of interest detected in each of the four fingerprints taken from three participants who each touched 2 mg heroin (Scenario 1). Despite the participants not having consumed heroin, all relevant analytes (including heroin metabolites) were detected. Our previous publication showed that heroin and 6-AM can be detected in fingerprints after administration. These data show for the first time that detection of heroin metabolites in a fingerprint collected from unwashed hands can also result from dermal contact (4).

**Figure 3:**
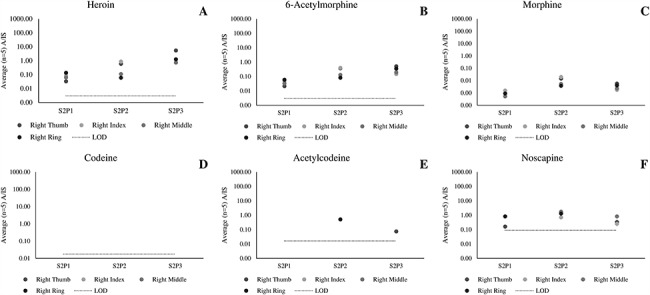
Average (*n* = 5 repeat injections) A/IS ratio of (A) heroin, (B) 6-acetylmorphone, (C) morphine, (D) codeine, (E) acetylcodeine and (F) noscapine measured for fingerprint samples collected after dermal contact with 2 mg of street heroin, wiping the fingerprints with alcohol-free wipes and wearing gloves for 10 minutes (Scenario 2).

Figure 3(A–E) presents data collected from participants who had touched 2 mg heroin, followed by hand wiping with alcohol-free wipes (Scenario 2). In this case, all analytes other than codeine and acetylcodeine are detected. Therefore hand wiping is insufficient to remove heroin, 6-AM, morphine or noscapine from a fingerprint following recent contact.

**Figure 4:**
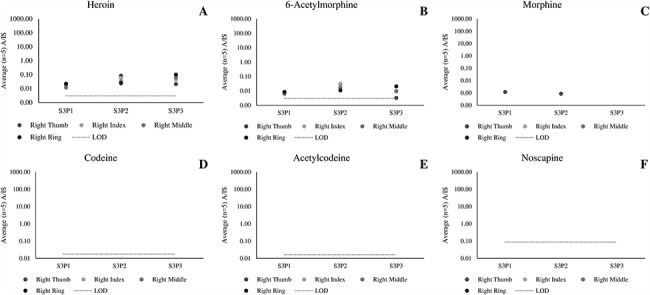
Average (*n* = 5 repeat injections) A/IS ratio of (A) heroin, (B) 6-acetylmorphone, (C) morphine, (D) codeine, (E) acetylcodeine and (F) noscapine measured for fingerprint samples collected after dermal contact with 2 mg of street heroin, washing hands with soap and wearing gloves for 10 minutes (Scenario 4).

Figure 4(A–E) presents data collected from participants who touched 2 mg heroin, followed by hand washing (Scenario 3). Here, heroin and 6-AM still continue to persist on the fingers. In contrast, codeine, acetylcodeine and noscapine were not observed. Morphine was detected in just 2 out of 12 fingerprints at low levels (1,000 times lower than in the samples collected in Scenario 1).

**Figure 5:**
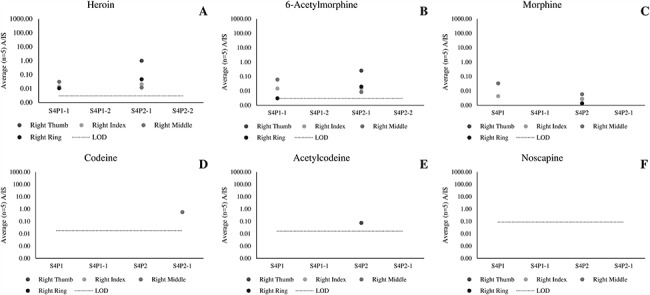
Average (*n* = 5 repeat injections) A/IS ratio of (A) heroin, (B) 6-acetylmorphone, (C) morphine, (D) codeine, (E) acetylcodeine and (F) noscapine measured using LC–HR-MS for fingerprint samples collected as part of a secondary transfer scenario (Scenario 4).

Figure 5(A–E) presents data collected from two participants who shook hands with a third participant who touched 2 mg heroin (S4P1–1 and S4P2–1), to explore secondary transfer. These participants were then asked to wash their hands and then provide a second set of fingerprint samples (S4P1–2 and S4P2–2). While the figure shows that heroin, 6-AM and morphine were transferred through the handshaking process, no relevant analytes were detected after hand washing.

Supplementary Data Table 2 present the oral fluid test results of 10 patients at a drug rehabilitation clinic, alongside whether they testified taking opiates in the last 24 hours. Only two patients, 41,028 and 41,026, testified not taking an opiate substance. These patients had a corresponding negative oral fluid test result. Three patients testified taking heroin but returned a negative oral fluid result. One patient (41,036) testified taking morphine only, confirmed by the oral fluid test result for this patient.

**Figure 6:**
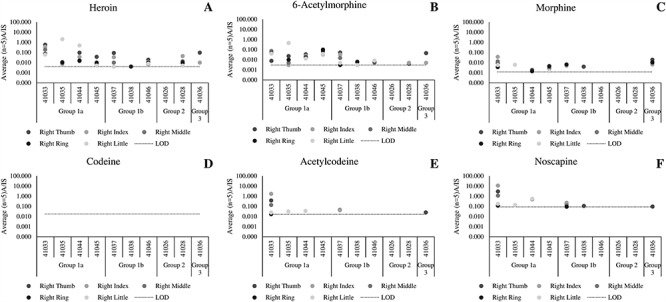
Average (*n* = 5 repeat injections) A/IS ratio of (A) heroin, (B) 6-acetylmorphine, (C) morphine, (D) codeine, (E) acetylcodeine and (F) noscapine measured using LC–HR-MS for samples collected from patients at a drug rehabilitation clinic after hand washing with soap and wearing gloves for 10 minutes.

Figure 6(A–E) presents data from the fingerprints of patients, collected after the patients washed their hands. The data are grouped according to the patient testimony on opiate use during the past 24 hours. Group 1 testified taking an opiate substance, whereas Group 2 testified not to have taken an opiate substance. Group 1 is then sub-divided into Group 1a (positive oral fluid test result), Group 1b (negative oral fluid test result) and Group 3 (morphine use only). The data for morphine are plotted in relation to a threshold of A/IS = 0.0012, which represents the highest signal obtained from a nondrug user after hand washing, to assure significance. Codeine was not detected in any patient sample. Morphine, acetylcodeine and noscapine were detected in a significant number of patient fingerprint samples, and this is discussed below.

## Discussion

The detection of drug *metabolites* from a contact only scenario (Scenario 1) has not been reported before. This finding shows that it is imperative that hands are cleaned prior to obtaining fingerprint samples if there is a need to make a distinction between heroin contact and heroin administration, particularly since it was not possible to find a ratio of parent drug:metabolite that could be used to distinguish the two donor groups.

The data from Scenario 4 show that the hand washing procedure adopted here is sufficient to remove residues incorporated in fingerprints through secondary transfer. This supports our previous observation that shaking hands with a drug user did not return a positive fingerprint test result for heroin, provided hands were washed prior to giving a fingerprint sample (4).

The data from Scenarios 2 and 3 show that hand washing or wiping is insufficient to remove all traces of heroin and 6-AM *directly* after contact with heroin. Furthermore, even though wiping or washing reduced the levels of analytes by over an order of magnitude, there was no statistically significant difference between the levels of analyte in fingerprints collected after contact or administration. This means that the detection of heroin and 6-AM alone is insufficient to distinguish between contact and administration of heroin, even after hand washing. In contrast, hand washing was sufficient for the removal of all traces of acetylcodeine and noscapine as well as all morphine signals with A/IS > 0.0012.

The patient data show that all patients in Group 1a (positive oral fluid test for heroin) donated at least one fingerprint containing traces of morphine, in addition to the expected heroin and 6-AM. Acetylcodeine and noscapine were less frequently detected than the other analytes. For the patients in Group 1b (positive testimony, negative oral fluid) morphine, acetylcodeine or noscapine were also detected in 2 out of 3 patients. As in previous work, we attribute this discrepancy between fingerprints and oral fluid to be due to a difference in detection window (i.e., how long an analyte is detectable in the sampling matrix *via* the detection method used).

Morphine, acetylcodeine or noscapine were not detected in the fingerprints of patients in Group 2, (testified not to have taken heroin), showing good agreement between oral fluid, fingerprint and patient testimony. In contrast, heroin and 6-AM were detected in the fingerprints of one of these patients (41,028), presumably through dermal contact with the substance or a contaminated surface. This result is consistent with the results of the contact residue experiment presented in Figure 4, where it was shown that heroin and 6-AM could be detected after contact with street heroin, even if donors wash their hands. In contrast, morphine, acetylcodeine and noscapine provide better agreement with patient testimony, presumably because contact residues can be washed off. Therefore we propose that future studies could focus on the relevance of the detection of morphine, acetylcodeine and noscapine in a fingerprint, to determine whether they can be used to distinguish between administration and dermal contact of heroin. A limitation of this approach is that the method lacked sensitivity to detect these analytes in *all* replicate fingerprints and therefore the sensitivity should be improved in future work, using a more targeted approach for these analytes.

While these data appear to show a suitable strategy for distinguishing between contact and administration of heroin, the patient in Group 3 shows that there is an alternative interpretation for a fingerprint containing all the analytes considered here. The patient in Group 3 testified taking morphine only. This is corroborated by the oral fluid result, and the detection of morphine in the fingerprints collected from this patient. However, for this patient, heroin and 6-AM were also detected, alongside acetylcodeine and noscapine. One possible explanation for this observation is that the patient’s testimony was incorrect, or that the traces thought to be relevant to heroin use originate from a previous heroin dose. An alternative explanation is that it is known that noscapine and codeine as well as other co-extracted alkaloids are present in street morphine (including a compound tentatively identified as acetylcodeine) (28). Therefore it is possible that the source of acetylcodeine and noscapine in the fingerprints of this patient arises from morphine administration. For other drug testing matrices, 6-AM can be used to distinguish between heroin administration and morphine use (29–31). In the case of a fingerprint, the detection of 6-AM implies *either* administration *or* recent contact with heroin. Morphine can be used to confirm that heroin has been ingested rather than touched, but future work is needed to confirm whether morphine and heroin administration can be distinguished from one another.

The data presented here only considers the detection of heroin and related compounds immediately after dermal contact with seized heroin. This was done as a worst-case scenario because levels would be highest immediately after contact. Of course it is possible that over time, the relative abundance of different analytes changes and this could be explored by future studies as an additional means to distinguish contact and administration scenarios.

## Conclusions

A fingerprint provides a convenient matrix for drug testing because samples can be given quickly and painlessly. Additionally, the ridge details that are embedded in the sample provide an opportunity for improved traceability and security. While our previous work has shown that heroin is not a common environmental contaminant on the fingers, this report is the first to study the difference in fingerprint samples collected after (i) deliberate contact and (ii) administration of heroin. Due to the fact that both heroin and its main metabolite (6-acetylmorphine) were not removed by hand washing after contact with heroin, the detection of these substances in fingerprint may not only be indicative of heroin administration. On the other hand, the detection of morphine, acetylcodeine and noscapine could be removed through hand washing and were detected in a significant percentage of the samples collected from patients at a drug rehabilitation clinic that testified taking heroin. Thus, if there is a question about the source of heroin found in a fingerprint, future studies should explore whether detection of morphine, acetylcodeine or noscapine can assist with the interpretation.

## Supplementary Material

jat-19-2870-File008_bkz088
