# Supplementary material for: Distinguishing between Contact and Administration of Heroin from a Single Fingerprint using High Resolution Mass Spectrometry
Source: J Anal Toxicol. 2019 Nov 4;44(3):218–25. doi: 10.1093/jat/bkz088 (PMC7299524; doi:10.1093/jat/bkz088)
Supplement: jat-19-2870-File008_bkz088 [file jat-19-2870-file008_bkz088.pdf]

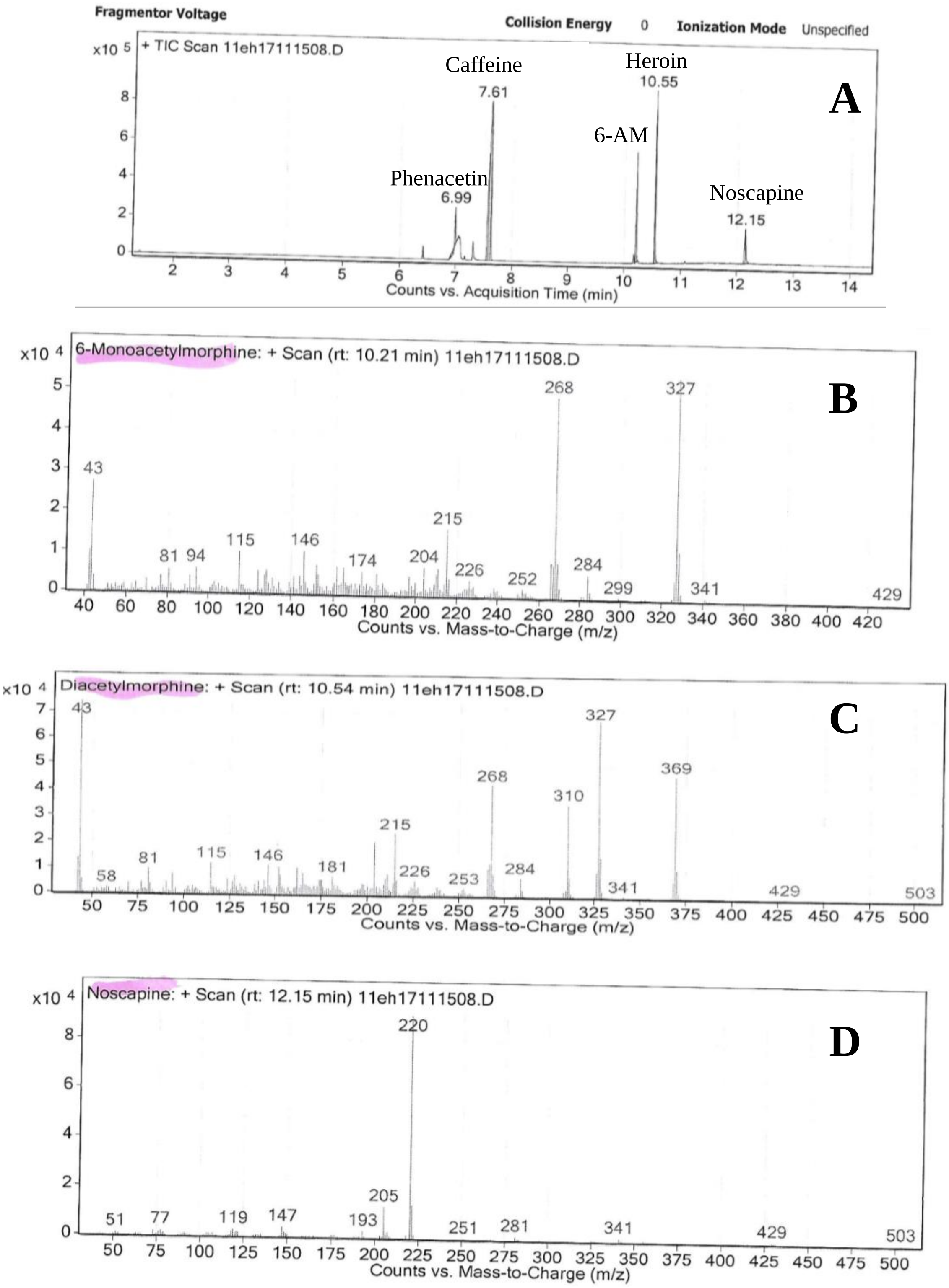

Supplemental Data Figure 1. Gas chromatography-mass spectrometry results by Forensic Science Ireland of seized heroin. (A) Total ion chromatogram (TIC) of heroin samples; (B) MS/MS spectrum of 6-monoacetylmorphine (6-AM) peak at retention time 10.21 min; (C) MS/MS spectrum of heroin (diacetylmorphine) peak at retention time 10.54 min; (D) MS/MS spectrum of noscapine peak at retention time 12.15 min.

Supplemental Data Table 1. Protonated molecular ion monoisotopic mass and respective retention times for morphine, codeine, acetylcodeine and noscapine.

**Substance**	**$[M+H]^+$**	**Retention time (min)**
**Morphine**	286.1427	1.62
**Codeine**	300.1594	1.78
**Acetylcodeine**	342.1700	2.05
**Noscapine**	414.1547	2.15

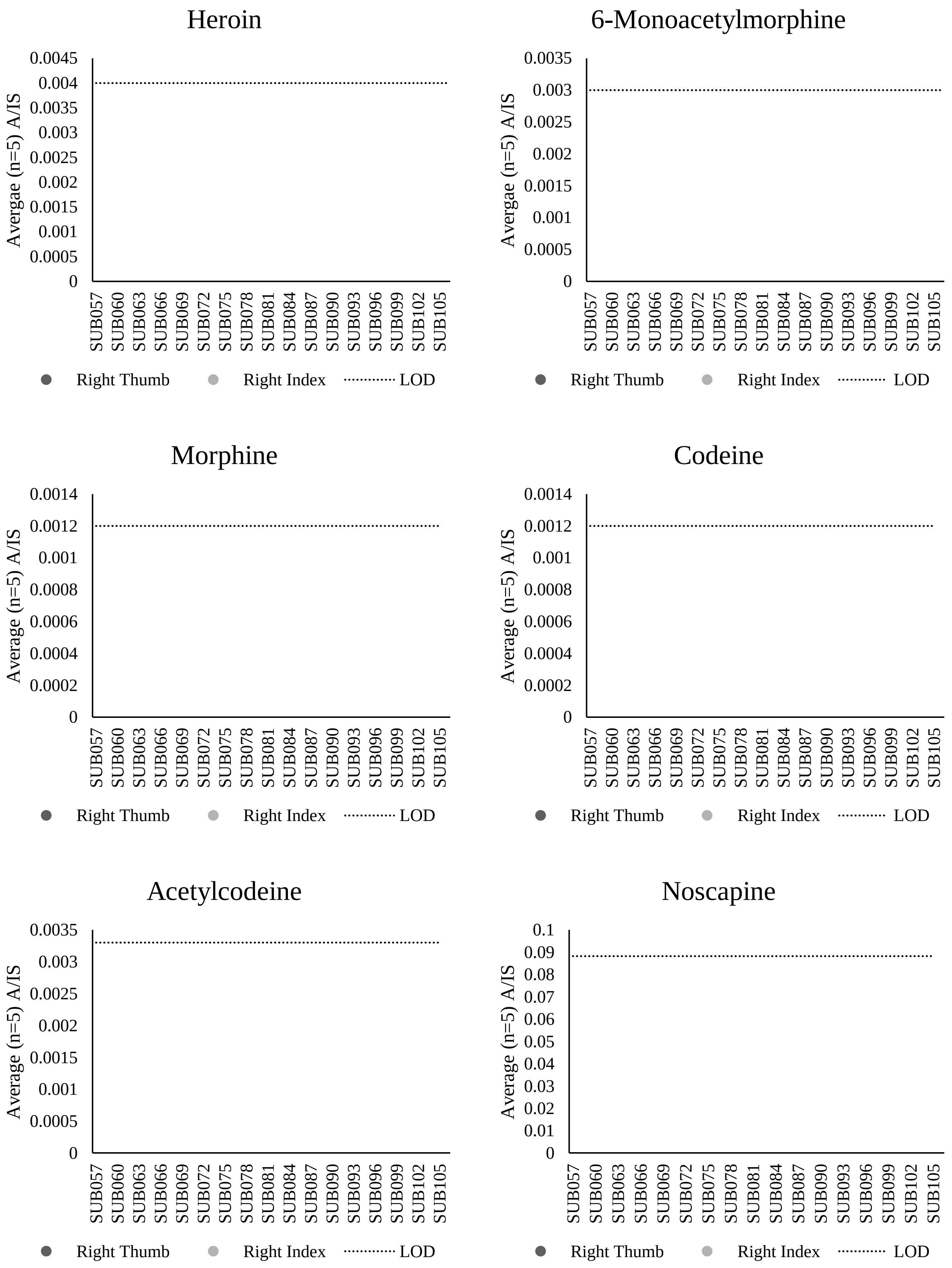

Supplemental Data Figure 2. Results from the analysis of fingerprint samples collected from 50 non-drug users after handwashing with soap using LC-MS.

Supplemental Data Table 2. Corresponding oral fluid results and patient testimony for fingerprint samples collected from patients at a drug rehabilitation clinic.

	**Participant #**	**Patient Testimony**	**Oral Fluid Results**
**Group 1a**	**41033**	Heroin	*Positive*<br>Morphine: >240 ng/mL<br>6-AM: >32 ng/mL<br>Codeine: 74 ng/mL
	**41035**	Heroin	*Positive*<br>Morphine: 138 ng/mL<br>6-AM: 32 ng/mL<br>Codeine: 36 ng/mL
	**41044**	Cocaine, Heroin, Cannabis	*Positive*<br>6-AM: 23 ng/mL
	**41045**	Crack cocaine, Heroin	*Positive*<br>Morphine: 90 ng/mL
**Group 1b**	**41037**	Heroin, Cocaine (crack), Diazepam	*Negative*
	**41038**	Heroin, Cocaine	*Negative*
	**41046**	Heroin, Cocaine	*Negative*
**Group 2**	**41026**	Cocaine	*Negative*
	**41028**	Cocaine	*Negative*
**Group 3**	**41036**	Cocaine, Morphine	*Negative*
